# Molecular Surveillance of *Neoehrlichia mikurensis* and *Anaplasma phagocytophilum* in Ticks from Urbanized Areas of Lithuania

**DOI:** 10.3390/pathogens14070642

**Published:** 2025-06-28

**Authors:** Justina Snegiriovaitė, Indrė Lipatova, Miglė Razgūnaitė, Algimantas Paulauskas, Jana Radzijevskaja

**Affiliations:** Faculty of Natural Sciences, Vytautas Magnus University, K. Donelaičio Str. 58, LT-44248 Kaunas, Lithuania; justina.snegiriovaite@vdu.lt (J.S.); indre.lipatova@vdu.lt (I.L.); migle.razgunaite@vdu.lt (M.R.); jana.radzijevskaja@vdu.lt (J.R.)

**Keywords:** *Ixodes ricinus*, *Dermacentor reticulatus*, tick-borne pathogens, zoonosis

## Abstract

*Neoehrlichia mikurensis* and *Anaplasma phagocytophilum*, both members of the Anaplasmataceae family, are pathogens that can cause diseases in animals and humans. Ixodid ticks are the primary vectors for both species. While urban green spaces offer various ecological and social benefits, there is increasing evidence suggesting potential public health risks, particularly increased exposure to vector-borne diseases. The aim of the present study was to assess the prevalence and co-occurrence of *A. phagocytophilum* and *N. mikurensis* in ticks from urban environments in Lithuania. A total of 3599 *Ixodes ricinus* and 29 *Dermacentor reticulatus* were collected from 31 urban and 21 peri-urban areas. Ticks were examined for pathogens using duplex real-time PCR. *Anaplasma phagocytophilum* was detected in 4.47% of tested ticks, while *N. mikurensis* in 6.17%. Co-infection was found in 0.42% of *I. ricinus* specimens. Phylogenetic analysis of the *groEl* gene revealed low genetic variability of *N. mikurensis* and the circulation of two ecotypes (I and II) of *A. phagocytophilum*. Additionally, *Ehrlichia muris* was identified in *I. ricinus* ticks. This study is the first report of *N. mikurensis* detection in ticks from Lithuania. Our findings highlight the potential risk posed by tick-borne pathogens in urban and peri-urban areas of the country.

## 1. Introduction

Ticks are competent vectors of a wide range of pathogens, including bacteria, viruses, and protozoa, which can cause various diseases in humans and animals [1]. Global warming is influencing the distribution and abundance of ticks, potentially increasing the risk of tick-borne diseases [2]. The two widely distributed tick species in Lithuania, *Ixodes ricinus* and *Dermacentor reticulatus*, are among the most abundant and epidemiologically significant tick species in Europe [3,4]. The prevalence and distribution of these ticks, as well as the pathogens they transmit, are influenced by ecological factors such as biotope type and climate conditions [5].

The family Anaplasmataceae is a group of small, Gram-negative, pleomorphic, obligate intracellular bacteria within the order Rickettsiales [6]. This family includes five genera: *Anaplasma*, *Ehrlichia*, *Neoehrlichia*, *Neorickettsia*, and *Wolbachia*. Members of Anaplasmataceae family infect various host cells, including erythrocytes, monocytes, macrophages, neutrophils, and platelets [7,8,9]. Ixodidae ticks serve as vectors for numerous Anaplasmataceae bacteria, which can cause disease in humans and a wide range of domestic and wild animals [10].

*Neoehrlichia mikurensis* is a newly identified tick-borne bacterium belonging to the family Anaplasmataceae [11]. This pathogen was first time reported in wild rats in Japan [12]. To date, its presence has been recorded in at least 20 European countries [13]. Ticks of the genus *Ixodes* are recognized as the primary vector of *N. mikurensis* [11,14]. Although the pathogen has also been detected in *Dermacentor* spp. ticks, the role of this tick genus in its transmission remains unclear [15]. Rodents are considered the main reservoir host, although *N. mikurensis* has also been identified in other wild and domestic animals [13].

*Anaplasma phagocytophilum* is another member of the Anaplasmataceae family, which causes human granulocytic anaplasmosis (HGA), tick-borne fever (TBF), and granulocytic infections in animals [16]. While wild ruminants and small mammals are thought to play a central role in maintaining *A. phagocytophilum* in nature, various other animals, such as bears, foxes, wild boars, horses, and reptiles, may also serve as occasional hosts or reservoir species [17]. *Anaplasma phagocytophilum* has a broad geographic distribution in Europe and exhibits significant genetic diversity, with different strains showing both host and regional specificity [10,18].

In Lithuania, *A. phagocytophilum* has previously been detected in questing ticks, ticks collected from migratory birds, cervids, and European bison, as well as in samples from domestic dogs, raccoon dogs, roe deer, and European bison [19,20,21,22,23,24]. However, there is a lack of data on the presence of *A. phagocytophilum* in ticks from urban environments. Furthermore, no studies have been conducted to investigate the presence of *N. mikurensis*. The aim of this study was to assess the prevalence and co-occurrence of *A. phagocytophilum* and *N. mikurensis* in ticks from urban environments in Lithuania.

## 2. Materials and Methods

### 2.1. Tick Collection and DNA Extraction

A total of 52 green spaces (31 urban and 21 peri-urban) across 22 cities and ten counties in Lithuania were selected for tick sampling (Table S1). Priority was given to heavily frequented recreational areas with suitable ecological conditions for ticks. Questing ticks were collected between April and June during the years 2021–2024 using standard flagging and dragging techniques. All ticks were identified to the species level, developmental stage, and sex using taxonomic keys [25] and were preserved in 70% ethanol.

Genomic DNA was extracted from individual tick specimens by lysis in 2.5% ammonium hydroxide solution [26]. The extracted DNA was then stored at −20 °C for subsequent molecular analyses.

### 2.2. PCR Assay and Sequencing

A duplex real-time PCR assay was used for the simultaneous detection of *A. phagocytophilum* and *N. mikurensis* in tick samples, targeting 98 bp and 129 bp fragments of the *msp2* and *groEL* genes, respectively [27,28]. Reactions were performed on a Rotor-Gene Q (Qiagen, Venlo, The Netherlands) using 2x Sensi Mix™ II Probe No-ROX (Bioline, London, UK). Nucleotide sequences of the primers and TaqMan probes used for the detection of *A. phagocytophilum* and *N. mikurensis* are listed in Table 1. Each 15 μL reaction mixture contained 2 μL of template DNA, 1x Sensi Mix™ II Probe No-ROX, 1 pM of each primer, and 0.5 pM of each probe. The thermal cycling conditions were as follows: initial denaturation at 95 °C for 2 min, followed by 50 cycles of denaturation at 95 °C for 20 s, annealing at 60 °C for 1 min, and extension at 72 °C for 20 s. Both negative and positive controls were included in each run. Positive controls consisted of *I. ricinus* tick DNA samples previously confirmed as positive for *A. phagocytophilum* and *N. mikurensis* by sequencing. Results were considered positive when repeats yielded cycle threshold (Ct) values < 38.

Positive *A. phagocytophilum* and *N. mikurensis* samples identified by real-time PCR were further confirmed using a nested PCR (nPCR) assay. Two primer sets, HS1a/HS6a and HS43/HSVR, were used in nPCR to amplify an approximately 1300 bp fragment of the *groEL* gene in species of the Anaplasmataceae family [29,30]. Each 25 μL reaction mixture for both PCR rounds contained 1x Dreamtaq Green Master Mix (ThermoFisher Scientific, Vilnius, Lithuania), 0.4 μM of each primer, nuclease-free water, and 2 µL of DNA template for the first round, or 1 µL of the first-round PCR product for the second round. The amplification program starts with three cycles of DNA denaturation at 94 °C for 1 min, annealing at 48 °C for 2 min, and extension at 70 °C for 1 min 30 s. This was followed by 37 cycles of denaturation at 88 °C for 1 min, annealing at 52 °C for 2 min in the first-round PCR and at 55 °C for 2 min in the second round. The final extension step was then carried out at 68 °C for 5 min, and the reaction was held at 10 °C.

PCR amplicons from *A. phagocytophilum* and *N. mikurensis* positive samples were excised from 1.5% agarose gels and purified using the GeneJET Gel Extraction Kit (Thermo Fisher Scientific, Vilnius, Lithuania) according to the manufacturer’s instructions. The purified products were sent for sequencing to Macrogen (Amsterdam, The Netherlands). The obtained sequences were edited, aligned with one another, and compared with the sequence from GenBank using the software Mega 11 version 11.0.3 [31] and the BLASTn algorithm. The phylogenetic tree for the *groEL* gene was constructed using the Maximum Likelihood method with the Tamura 3-parameter model and bootstrap analysis with 1000 replicates.

The sequences obtained in this study were submitted to the GenBank database under the accession numbers PV711389–PV711402 for *A. phagocytophilum* and PV711403–PV711412 for *N. mikurensis*.

### 2.3. Statistical Analysis

A statistical analysis was performed using Statistica for Windows (version 7.0, StatSoft, Tulsa, OK, USA). A chi-square test and 95% confidence intervals (95% CI) were used to compare differences in the prevalence of detected pathogen species across tick species, development stages, sex, and the area types. Differences were considered statistically significant when *p* < 0.05.

## 3. Results

### 3.1. Prevalence of A. phagocytophilum and N. mikurensis

A total of 3628 ticks were collected from 43 out of 52 urban and peri-urban locations. The ticks were morphologically identified as *I. ricinus* (*n* = 3599) and *D. reticulatus* (*n* = 29) (Table 2). *Anaplasma phagocytophilum* was detected in 4.47% of tested tick samples (162/3628) (95% CI: 3.79–5.14), while *N. mikurensis* was found in 6.17% (224/3628) (95% CI: 5.39–6.96). Among the identified tick species, only one *D. reticulatus* female (3.45%) was found to be infected with *A. phagocytophilum*, while no specimens were positive for *N. mikurensis*. In contrast, infections with both pathogens were predominantly detected in *I. ricinus*, which showed significantly higher prevalence rates (4.47% for *A. phagocytophilum* and 6.22% for *N. mikurensis*). No substantial differences in infection rates were observed between urban and peri-urban areas for either pathogen species: *A. phagocytophilum* was detected in 4.63% (95% CI: 3.95–5.32) of ticks from urban areas and 4.31% (95% CI: 3.64–4.97) from peri-urban areas; *N. mikurensis* was found in 5.87% (95% CI: 5.11–6.64) and 6.46% (95% CI: 5.66–7.26), respectively. Similarly, infection rates by habitat type showed little variation, with *A. phagocytophilum* prevalence at 4.53% (95% CI: 3.85–5.20) in forested sites and 4.35% (95% CI: 3.69–5.02) in non-forested areas, while *N. mikurensis* was found in 5.85% (95% CI: 5.09–6.61) and 6.76% (95% CI: 5.95–7.58), respectively. However, infection prevalence varied more substantially across individual sampling locations, ranging from 0.71% to 23.53% for *A. phagocytophilum* and from 0.87% to 16.67% for *N. mikurensis* (Table S1).

A total of 15 cases of co-infection (0.42%; 95% CI: 0.21–0.63) with *A. phagocytophilum* and *N. mikurensis* were detected in *I. ricinus* ticks. Co-occurrence of both pathogens was significantly more frequent in males than females (χ^2^ = 4.83, *p* = 0.03), and in adult ticks compared to nymphs (χ^2^ = 0.21, *p* = 0.65). Co-infections were more commonly observed in peri-urban sites (10 cases) than in urban ones (5 cases) (χ^2^ = 1.38, *p* = 0.24). Similarly, the vast majority of co-infection cases (13 out of 15) were detected in forested habitats (χ^2^ = 3.76, *p* = 0.05) (Table 2).

### 3.2. Phylogenetic Analysis

**Figure 1:**
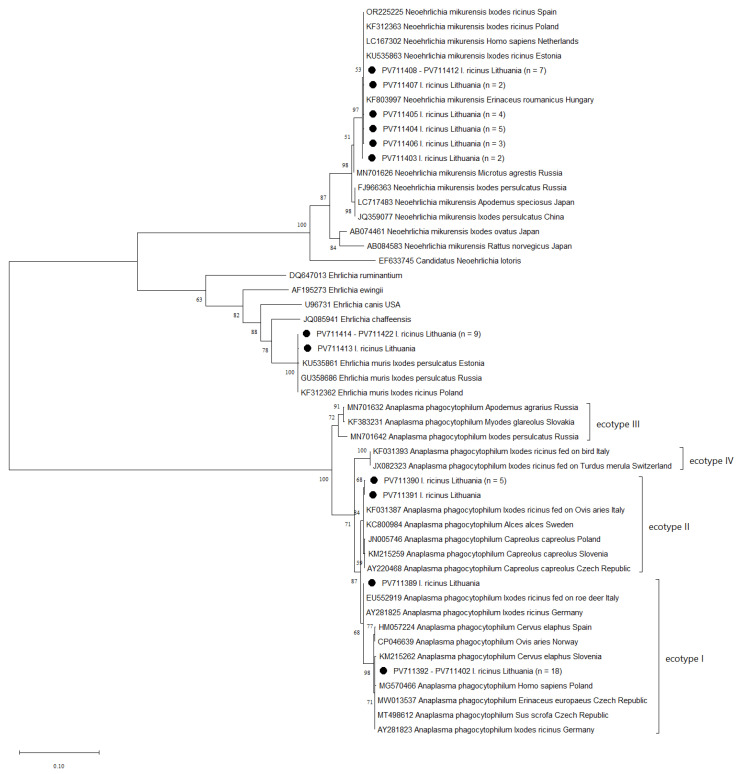
Maximum-likelihood phylogenetic tree based on partial *groEL* gene sequences. Samples isolated from ticks in this study are marked with ●. The number of samples represented by the sequence is given in parentheses (*n* = x).

Analysis of the obtained partial *groEL* gene sequences revealed that *I. ricinus* ticks were infected with three pathogen species: *A. phagocytophilum*, *N. mikurensis*, and *Ehrlichia muris* (Figure 1). The *A. phagocytophilum* sequences showed 99.9–100% identity to reference sequences deposited in GenBank. Most of the sequences from this study clustered within ecotype I, while a few were assigned to ecotype II. Four distinct sequence variants of the *A. phagocytophilum groEL* gene were identified, with 19 variable nucleotide positions. The Lithuanian *N. mikurensis* sequences were 99.8–100% identical to *N. mikurensis* sequences from other European countries. In this study, six sequence variants of the *N. mikurensis groEL* gene with six variable nucleotide positions were identified, differing by one to six nucleotides among the identified variants. Moreover, ten of the sequences matched *E. muris* reference sequences in GenBank with 99.9–100% identity, supporting their classification as *E. muris*. Sequence comparison revealed two *E. muris* variants, differing by a single nucleotide substitution (G→C).

## 4. Discussion

To the best of the authors’ knowledge, this is the first report on the detection of *N. mikurensis* in ticks from Lithuania. *N. mikurensis* was found in *I. ricinus* ticks collected from both urban and peri-urban areas. This study confirmed the presence of *A. phagocytophilum* in *I. ricinus* and *D. reticulatus* ticks from urbanized environments, extending previous findings that had primarily identified this pathogen in ticks from natural habitats in Lithuania [19,21].

In Lithuania, Lyme borreliosis and tick-borne encephalitis are the only tick-borne diseases routinely diagnosed in humans. Other potential tick-borne diseases, such as anaplasmosis, babesiosis, ehrlichiosis, and rickettsiosis, which can cause flu-like symptoms, are not investigated in clinical practice. In comparison, HGA has been clinically confirmed and verified through laboratory testing in Austria, Italy, Poland, and several other European countries [32]. Our study demonstrates the presence of *A. phagocytophilum* and *N. mikurensis* in frequently visited public green spaces in Lithuania. Therefore, expanding the list of tick-borne diseases included in medical surveillance and diagnostics is essential for improved disease recognition and management.

In this study, the overall prevalence of *A. phagocytophilum* was 4.47%. By comparison, studies from Poland, Latvia, and Estonia reported significantly lower prevalence rates (1.7%, 1.1%, and 0.5%, respectively) [33,34,35]. *Anaplasma phagocytophilum* has been identified in multiple animal species, including both domestic and wild mammals. A recent global meta-analysis reported a 15.18% prevalence of *A. phagocytophilum* in animal reservoirs, with an infection rate approximately twice as high in wildlife compared to domestic animals, highlighting the importance of wild hosts in the maintenance and transmission of this pathogen [36]. In Europe, *A. phagocytophilum* is divided into four main ecotypes based on *groEL* gene sequences [37]. Sequences obtained in this study from ticks collected in urban and peri-urban areas belonged to ecotype I and ecotype II, with ecotype I being predominant. Ecotype I has been detected in a wide range of hosts and is associated with HGA and TBF. In contrast, ecotype II has primarily been found in roe deer and moose and is currently considered non-zoonotic [37,38]. The predominance of ecotype I in tick samples from urban and peri-urban areas highlights a potential risk of infection, particularly among people and pets spending time in urban green spaces.

The detection of *N. mikurensis* at an overall prevalence rate of 6.22% highlights its established circulation in urban tick populations in Lithuania, suggesting a potential risk of human infection. The prevalence varied considerably across different sampling locations within the country, ranging from 0.87% to 16.67%, indicating spatial heterogeneity in pathogen distribution. The prevalence of *N. mikurensis* also varies among Lithuania’s neighboring countries, with lower rates reported in Estonia (2.4%) and higher rates in Poland (19.3%) [9,39]. Rodents are important reservoir hosts for *N. mikurensis*, playing a key role in its maintenance and circulation in both urban and natural habitats [40]. In this study, *N. mikurensis* was detected only in *I. ricinus* ticks. A similar observation was reported in studies from Germany and Poland, where *D. reticulatus* tested negative for this pathogen [5,41]. Furthermore, previous studies have reported low genetic diversity in *N. mikurensis groEL* gene sequences across various European countries [28,42,43]. Similarly, our phylogenetic analysis of the *groEL* gene sequences revealed limited genetic variation among the strains detected in this study. The low genetic variability of *N. mikurensis* likely results from its adaptation to a narrow ecological niche, involving a limited range of reservoir hosts and vectors [43]. No clear evidence currently links genetic variation with differences in *N. mikurensis* pathogenicity. This pathogen often causes mild or asymptomatic infections, especially in immunocompetent individuals [44]. Further research is necessary to clarify its pathogenic potential.

In this study, *E. muris* was incidentally detected in several *I. ricinus* tick samples. The detection is consistent with previous reports, which showed that *groEL* gene primers also amplify non-target *Ehrlichia* species [29,45]. *Ehrlichia muris* primarily affects various rodent species but can also infect humans [46,47,48]. It was also reported from dogs [49]. Hard tick species, such as *Ixodes persulcatus* and *I. ricinus* in Eurasia, and *Ixodes scapularis* in the USA, are suggested as a potential vectors for this pathogen [48,50]. Although the prevalence of *E. muris* was not systematically assessed in our study, its incidental detection may indicate the presence of co-infection with the target pathogen. Further targeted investigations would be necessary to confirm such co-infections and evaluate their epidemiological significance.

In this study, co-infection with *A. phagocytophilum* and *N. mikurensis* was also detected. Ticks harboring more than one pathogen represent a potential source of co-infection for both humans and animals [51]. Co-occurrence of *N. mikurensis* and *A. phagocytophilum* has previously been reported in *I. ricinus* ticks from Central Europe [11]. In Poland, a significantly higher occurrence of Anaplasmataceae species co-infections was observed in *I. ricinus* ticks from urban areas compared to natural habitats [52]. These pathogens are known to share similar mammalian reservoir hosts, such as rodents, and it has been suggested that they may facilitate each other’s transmission by ticks, as shown for *A. phagocytophilum* and *Borrelia* spp. [52,53]. However, further studies are needed to confirm this interaction.

## 5. Conclusions

The present findings confirm the presence of tick-borne pathogens in urban and peri-urban areas of Lithuania. Although no human cases of anaplasmosis, neoehrlichiosis, or ehrlichiosis have been officially registered in Lithuania so far, these infections are likely underdiagnosed or misclassified in clinical practice as other flu-like illnesses. Therefore, expanding diagnostic awareness and integrating these pathogens into routine surveillance is warranted.

## Figures and Tables

**Table 1:** Nucleotide sequences of primers and probes used in this study.

Primers and Probes	Sequences (5′-3′)	Target Gene	Amplicon Size	References
Anaplasma_F	GGACAACATGCTTGTAGCTATGGAAGG	*msp2*	98 bp	[26]
Anaplasma_R	CCTTGGTCTTGAAGCGCTCGTA			
Anaplasma_Zr	VIC-TCTCAAGCTCAACCCTGGCACCACCA-BHQ1			
Neo2f	GCAAATGGAGATAAAAACATAGGTAGTAAA	*groEL*	129 bp	[27]
Neo2r	CATACCGTCAGTTTTTTCAACTTCTAA			
Neo2m	Cy5-TTACAGTTGAGGAAAGTAAGGGA-BHQ2			
HS1	AITGGGCTGGTAITGAAAT	*groEL*	1450 bp	[28,29]
HS6a	CCICCIGGIACIAIACCTTC			
HS43	ATWGCWAARGAAGCATAGTC		1300 bp	
HSVR	CTCAACAGCAGCTCTAGTAGC			

**Table 2:** Number of collected ticks and prevalence of *Anaplasma phagocytophilum* and *Nehrlichia mikurensis*. N—number of collected ticks; n—number of infected ticks.

		N	Prevalence, n (%) (95% CI)		Pathogen Co-Occurrence
			*Anaplasma phagocytophilum*	*Neoehrlichia mikurensis*	
Tick species	*Ixodes ricinus*	3599	161 (4.47) (3.80–5.15)	224 (6.22) (5.44–7.01)	15
	*Dermacentor reticulatus*	29	1 (3.45) (2.85–4.40)	0	0
Tick sex	Female	985	53 (5.38) (4.65–6.11)	66 (6.70) (5.89–7.51)	1
	Male	1040	65 (6.25) (5.46–7.04)	69 (6.63) (5.82–7.44)	8
Tick stage	Adults	2025	118 (5.83) (5.06–6.59)	135 (6.66) (5.85–7.48)	9
	Nymphs	1603	44 (2.74) (2.21–3.27)	89 (5.55) (4.81–6.29)	6
Area type	Urban	1770	82 (4.63) (3.95–5.32)	104 (5.87) (5.11–6.64)	5
	Peri-urban	1858	80 (4.31) (3.64–4.97)	120 (6.46) (5.66–7.26)	10
Habitat type	Forested	2342	106 (4.53) (3.85–5.20)	137 (5.85) (5.09–6.61)	13
	Non-forested	1286	56 (4.35) (3.69–5.02)	87 (6.76) (5.95–7.58)	2

## Data Availability

Data is contained within the article.

## Supplementary Materials

The following supporting information can be downloaded at: https://www.mdpi.com/article/10.3390/pathogens14070642/s1, Table S1: Tick collection sites, number of collected ticks and prevalence of *A. phagocytophilum* and *N. mikurensis*.
