# Supplementary material for: Molecular Surveillance of Neoehrlichia mikurensis and Anaplasma phagocytophilum in Ticks from Urbanized Areas of Lithuania
Source: Pathogens. 2025 Jun 28;14(7):642. doi: 10.3390/pathogens14070642 (PMC12298857; doi:10.3390/pathogens14070642)
Supplement: Supplementary file 1 [file pathogens-14-00642-s001.zip › pathogens-3707442-supplementary.pdf]

Table S1: Tick collection sites, number of collected ticks and prevalence of *A. phagocytophilum* and *N. mikurensis*

Area type	Coordinates	Site name	N	*A. phagocytophilum*		*N. mikurensis*		Co-infection	*A. phagocytophilum*, n / *N. mikurensis*, n / Total number of tested ticks, N				
									Ixodes ricinus			*Dermacentor reticulatus*	
				n	%	n	%		F	M	N	F	M
**Vilnius County**													
UF	54°40'53.3"N 25°14'07.6"E	Vingis Park, Vilnius	269	16	5.95	22	8.18	4	6/3/73	2/10/50	8/9/146	-	-
PUF	54°43'28.1"N 25°15'52.0"E	Fabijoniškės Park, Vilnius	251	14	5.58	16	6.37	2	5/6/49	7/3/70	2/7/132	-	-
UF	54°40'54.2"N 25°12'10.7"E	Fairytale Park, Vilnius	251	15	5.98	5	1.99	0	8/1/49	6/3/70	1/1/132	-	-
UF	54°42'57.3"N 25°14'5.02E"	Viršuliškės Forest Park, Vilnius	1	0	-	0	-	0	-	-	0/0/1	-	-
U	54°42'47.9"N 25°13'21.5"E	Lūžiai Park, Vilnius	115	13	11.30	1	0.87	0	4/0/13	3/0/19	6/1/83	-	-
PU	54°40'22.4N" 25°19'30.1E"	Markučiai Park, Vilnius	59	1	1.69	2	3.39	0	0/0/13	1/1/16	0/1/30	-	-
		**Subtotal**	**946**	**59**	**6.24**	**46**	**4.86**	**6**	**23/10/197**	**19/17/225**	**17/19/524**	**-**	**-**
**Kaunas County**													
U	54°52'10.4"N, 23°54'28.5"E	Botanical Garden, Kaunas	301	13	4.32	31	10.30	0	5/7/93	7/11/105	1/13/103	-	-
UF	54°52'34.3"N 23°58'01.1"E	Panemunė Forest Park, Kaunas	172	6	3.49	6	3.49	0	4/3/62	2/1/41	0/2/69	-	-
PUF	54°56'16.3"N 23°55'28.8"E	Kleboniškis Forest Park, Kaunas	407	21	5.16	42	10.32	4	1/9/89	10/12/75	10/21/243	-	-
U	54°53'54.8"N 23°56' 2.7"E	Oak Grove, Kaunas	2	0	-	0	-	0	-	0/0/2	-	-	-
U	54°53'50.3"N, 23°52'47.3"E	Confluence Park, Kaunas	0	-	-	-	-	-	-	-	-	-	-
PU	54°50'59.89"N 23°54'23.29"E	III Fort of Kaunas Fortress, Seniava, Kaunas district	27	0	-	0	-	0	0/0/2	0/0/11	0/0/13	0/0/1	0
PUF	54°51'54.23"N 24°25'39.12"E	Kaišiadorys Forest Park, Kiašiadorys	119	1	0.84	5	4.20	0	0/0/19	1/0/24	0/5/76	-	-
U	54°36'46.3"N 24°01'37.1"E	Central Park, Birštonas	174	8	4.60	11	6.32	0	5/7/91	3/4/81	0/0/2	-	-
PUF	54°34'38.77"N 23°57'4.82"E	Nemunas Loops Regional Park, Prienai district	99	7	7.07	7	7.07	1	1/3/23	2/3/27	4/1/49	-	-
PU	55°16'29.89"N 23°58'37.18"E	Green Area near the Cemetery, Kėdainiai	54	2	3.70	9	16.67	0	1/6/28	1/3/24	0/0/2	-	-
		**Subtotal**	**1355**	**58**	**4.28**	**111**	**8.19**	**5**	**17/35/407**	**26/34/390**	**15/42/557**	**0/0/1**	**0**
**Klaipėda County**													
U	55°40'49.24"N 21°10'53.4"E	Friendship Park, Klaipėda	0	-	-	-	-	-	-	-	-	-	-
U	55°43'3.31"N 21°8'24.37"E	Sculpture Park, Klaipėda	0	-	-	-	-	-	-	-	-	-	-
U	55°53'55.48"N 21°15'1.07"E	Manor Park, Kretinga	0	-	-	-	-	-	-	-	-	-	-
PU	55°49'58.39"N 21°24'4.85"E	Baubliai Park, Kretinga district	4	0	-	1		0	0/0/1	0/1/2	-	0/0/1	0
U	55°54'32.62"N 21°3'24.46"E	Birutė Park, Palanga	0	-	-	-	-	-	-	-	-	-	-
PUF	55°53'8.16"N 21°3'28.2"E	Seaside Regional Park, Palanga	141	1	0.71	10	7.09	0	0/0/16	1/3/18	0/7/107	-	-
		**Subtotal**	**145**	**1**	**0.69**	**11**	**7.58**	**0**	**0/0/17**	**1/4/20**	**0/7/107**	**0/0/1**	**0**
**Šiauliai County**													
U	55°55'9.24"N 23°16'18.99"E	Song Park, Šiauliai	158	4	2.53	14	8.86	0	0/5/44	1/2/49	3/7/65	-	-
PUF	55°55'49.1"N 23°21'2.83"E	Salduvė Park, Šiauliai	262	14	5.34	5	1.91	1	5/3/113	5/2/104	4/0/45	-	-
		**Subtotal**	**420**	**18**	**4.28**	**19**	**4.52**	**1**	**5/8/157**	**6/4/153**	**7/7/110**	**0**	**0**
**Panevėžys County**													
U	55°44'0.64"N 24°22'33.98"E	Skaistakalnis Park, Panevėžys	40	1	2.50	3	7.50	0	0/0/10	0/0/6	1/3/24	-	-
PUF	55°44'22.9"N 24°14'29.5"E	Berčiūnai Forest Park, Panevėžys	260	6	2.31	15	5.77	1	0/7/59	4/4/74	2/4/127	-	-
PU	55°30'32.08"N 24°18'40.43"E	Garuckai, Panevėžys district	17	4	23.53	1	5.88	1	2/0/3	2/1/14	-	-	-
		**Subtotal**	**317**	**11**	**3.47**	**19**	**5.99**	**2**	**2/7/72**	**6/5/94**	**3/7/151**	**0**	**0**
**Alytus County**													
U	54°23'39.13"N 24°2'51.68"E	City Park, Alytus	2	0	-	0	-	0	-	-	0/0/2	-	-
U	54°23'40.27"N 24°3'6.15"E	Resort Park, Alytus	23	2	8.69	3	13.04	1	0/1/11	2/1/6	0/1/6	-	-
U	54°1'14.9"N 23°58'41.52"E	Physiotherapy Park, Druskininkai	0	-	-	-	-	-	-	-	-	-	-
U	54°0'34.7"N 23°58'49.02"E	K. Dineika Wellness Park, Druskininkai	11	0	-	0	-	0	0/0/6	0/0/2	0/0/3	-	-
U	54°5'57.53"N, 23°41'23.87"E	Manor Park, Veisiejai	0	-	-	-	-	-	-	-	-	-	-
		**Subtotal**	**36**	**2**	**5.55**	**3**	**8.33**	**1**	**0/1/17**	**2/1/8**	**0/1/11**	**0**	**0**
**Marijampolė County**													
U	54°34'15.46"N 23°21'8.91"E	Pašešupys Park, Marijampolė	1	0	-	0	-	0	0/0/1	-	-	-	-
PU	54°32'49.5"N 23°19'56.29"E	Bear Mound, Marijampolė	11	0	-	0	-	0	0/0/2	-	0/0/9	-	-
U	54°38'38.77"N 23°2'46.5"E	Vilkaviškis Park, Vilkaviškis	8	0	-	0	-	0	0/0/1	-	0/0/7	-	-
PU	54°57'9.76"N 23°10'43.9"E	Good Ideas Park, Lukšiai, Šakiai district	30	0	-	0	-	0	0/0/4	0/0/8	0/0/18	-	-
		**Subtotal**	**50**	**0**	**-**	**0**	**-**	**0**	**0/0/8**	**0/0/8**	**0/0/34**	**0**	**0**
**Utena County**													
PUF	55°35'55.56"N 25°2'23.33"E	Adroniškis Park, Anykščiai district	27	3	11.11	2	7.41	0	0/1/7	3/1/14	-	0/0/4	0/0/2
U	55°44'10.15"N 26°13'41.56"E	The Great Island of Zarasas Lake, Zarasai	27	2	7.41	0	-	0	1/0/12	1/0/10	-	0/0/2	0/0/3
PU	55°48'23.96"N 25°55'47.85"E	Bright People's Park, Antazavė	52	1	1.92	2	3.85	0	1/2/22	0/0/21	0/0/1	0/0/1	0/0/7
PU	55°49'18.94"N 25°53'55.35"E	Marimont Water Mill, Zarasai district	27	1	3.70	0	-	0	0/0/9	0/0/12	-	1/0/4	0/0/2
U	55°44'41.28"N 25°50'12.99"E	Sculpture Park, Dusetos	20	0	-	0	-	0	0/0/2	0/0/15	0/0/3	-	-
PU	55°45'24.06"N 26°1'30.49"E	Aviliai II Park, Zarasai district	5	0	-	0	-	0	0/0/3	0/0/2	-	-	-
U	55°29'54.13"N 25°35'53.3"E	City Park, Utena	8	0	-	1	12.50	0	0/0/2	0/1/4	-	0	0/0/2
U	55°57'20.05"N 25°36'11.07"E	Lake Island, Rokiškis	7	0	-	1	14.28	0	0/0/4	0/1/3	-	-	-
		**Subtotal**	**173**	**7**	**4.05**	6	**3.47**	**0**	**2/3/61**	**4/3/81**	**0/0/4**	1/0/11	**0/0/16**
**Telšiai County**													
U	55°44'36.02"N 22°22'22.39"E	City Park, Varniai	0	-	-	-	-	-	-	-	-	-	-
PU	55°55'12.2"N 22°23'25.42"E	Pakapiai Recreation Area, Telšiai district	38	2	5.26	1	2.63	0	2/0/10	0/1/18	0/0/10	-	-
U	55°58'35.17"N 22°15'26.22"E	Culture Park, Telšiai	0	-	-	-	-	-	-	-	-	-	-
PUF	55°53'51.72"N 22°27'20.16"E	"The fairy's foot", Biržuvėnai,Telšiai district	26	1	3.85	0	-	0	0/0/1	0/0/2	1/0/23	-	-
PUF	55°58'10.99"N 22°10'25.62"E	Ilgis Walking Trail, Telšiai district	57	1	1.75	2	3.51	0	1/2/19	0/0/18	0/0/20	-	-
U	56°17'38.38"N 22°20'43.61"E	Juodpelkis Park, Mažeikiai	58	2	3.45	6	10.34	0	0/0/4	1/0/5	1/6/49	-	-
		**Subtotal**	**179**	**6**	**3.35**	**9**	**5.03**	**0**	**3/2/34**	**1/1/43**	**2/6/102**	**0**	**0**
**Tauragė County**													
U	55°15'5.37"N, 22°16'49.48"E	Jūra River Park, Tauragė	3	0	-	0	-	0	0/0/1	0/0/2	-	-	-
U	55°8'15.62"N 21°54'29.65"E	Town Park, Pagėgiai	4	0	-	0	-	0	0/0/1	-	0/0/3	-	-
		**Subtotal**	**7**	**0**	**-**	**0**	**-**	**0**	**0/0/2**	**0/0/2**	**0/0/3**	**0**	**0**
		**Total**	**3628**	**162**	**4.46**	**224**	**6.17**	**15**	**52/66/972**	**65/69/1024**	**44/89/1603**	**1/0/13**	**0/0/16**

Abbrevations:

N - total number of tested ticks
n - number of infected ticks
F - female
M - male
N - nymph
U - urban
UF - urban forest
PU - peri-urban
PUF - peri-urban forest
